# Platelet Levels of High- and Mega-Dose Methylprednisolone Treatment in Acute Immune Thrombocytopenia

**DOI:** 10.4274/tjh.2014.0436

**Published:** 2015-05-08

**Authors:** Ali Ayçiçek

**Affiliations:** 1 Kanuni Sultan Süleyman Research and Education Hospital, Clinic of Pediatric Hematology/Oncology, İstanbul, Turkey

**Keywords:** Immune thrombocytopenic purpura, Methylprednisolone, Glucocorticoids, child, adolescent

## TO THE EDITOR

High-dose methylprednisolone (HDMP) therapy was originally used by Ozsoylu et al. in the treatment of immune thrombocytopenic purpura (ITP), and it is also important in the history of Turkish hematology [1,2]. 

A 16-year-old male (weight: 107.5 kg, height: 167 cm, BMI: 38.5 kg/m2) presented with the complaints of oral submucosal bleeding and purpura. Complete blood count analysis revealed a platelet count of 1900/mm3, and his peripheral blood smear and bone marrow aspiration slides were consistent with ITP. Informed consent was obtained for HDMP and MDMP treatment.

High-dose methylprednisolone was initiated at a dose of 1000 mg/day for 3 days followed by 750 mg/day for 4 days, with each dose administered orally early in the morning. Platelet count reached 28,000/mm3 and 31,000/mm3 on the 3rd and 7th days of  treatment, respectively. Mucosal and cutaneous bleeding ceased. Methylprednisolone treatment was continued at a dose of 1 mg/kg/day for 7 days and tapered over a week. Platelet count was 22,000/mm3 on the last day, but bleeding reoccurred 10 days later and platelet count was 3900/mm3. 

**Figure 1:**
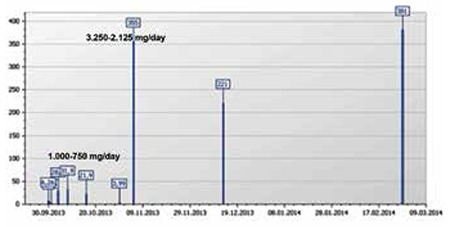
Figure 1. Platelet levels during high- and mega-dose methylprednisolone treatment (platelet count x 10^3^/mm^3^).

Mega-dose methylprednisolone (MDMP) was then given at a dose of 30 mg/kg (3250 mg/day) for 3 days, and then 20 mg/kg (2125 mg/day) for 4 days orally in a single dose in the early morning. Platelet count reached 355,000/mm3, 225,000/mm3, and 381,000/mm3 after 3, 35, and 100 days, respectively (Figure 1). No corticosteroid side effects were observed in this patient [3]. There was no thrombocytopenic attack in the last 12 months. 

HDMP treatment has been used as a therapeutic choice in childhood acute ITP in Turkey for a long time [2,4,5]. The term ‘HDMP’ was used for this kind of methylprednisolone administration initially, but it was later changed to MDMP since ‘HDMP’ was also used for 4-10 mg/kg doses in the literature [6]. It is reported that MDMP treatment differs from conventional corticosteroid (2 mg/kg in divided doses) and pulse methylprednisolone (1000 mg methylprednisolone infusion in 4 h) administration, not only by dose (30-100 mg/kg initially for 3 days, tapered gradually) but also the time of administration [7,8]. The doses (30 mg/kg for 3 days and 20 mg/kg for 4 days) of methylprednisolone for acute ITP treatment were also originally stated by Özsoylu and Ertürk [6]. 

Acute ITP is usually a benign, self-limited condition that occurs in young children, typically those younger than 10 years. In the majority of these patients, the thrombocytopenia resolves within weeks or a few months of the original manifestation. Glucocorticoid therapy in symptomatic childhood ITP patients has been suggested as an appropriate first-line therapy [2]. Therefore, I think that the results obtained with every high-dose corticosteroid cannot be compared. Based on previous experiences, I wish to conclude that MDMP is a cost-effective treatment for acute ITP, if treatment is required [7].

I would specifically like to emphasize MDMP treatment for these patients, which seems to be the most effective approach in the treatment of this disorder regardless of patient weight. In addition to some reports on intravenous immunoglobulin [9,10,11], MDMP is cheap, safe, and easily applicable in all conditions.
